# The Involvement of Ubiquitination Machinery in Cell Cycle Regulation and Cancer Progression

**DOI:** 10.3390/ijms22115754

**Published:** 2021-05-27

**Authors:** Tingting Zou, Zhenghong Lin

**Affiliations:** School of Life Sciences, Chongqing University, Chongqing 401331, China; tingtzou1997@163.com

**Keywords:** cell cycle regulation, CDKs, cyclins, CKIs, UPS, E3 ubiquitin ligases, Deubiquitinases (DUBs)

## Abstract

The cell cycle is a collection of events by which cellular components such as genetic materials and cytoplasmic components are accurately divided into two daughter cells. The cell cycle transition is primarily driven by the activation of cyclin-dependent kinases (CDKs), which activities are regulated by the ubiquitin-mediated proteolysis of key regulators such as cyclins, CDK inhibitors (CKIs), other kinases and phosphatases. Thus, the ubiquitin-proteasome system (UPS) plays a pivotal role in the regulation of the cell cycle progression via recognition, interaction, and ubiquitination or deubiquitination of key proteins. The illegitimate degradation of tumor suppressor or abnormally high accumulation of oncoproteins often results in deregulation of cell proliferation, genomic instability, and cancer occurrence. In this review, we demonstrate the diversity and complexity of the regulation of UPS machinery of the cell cycle. A profound understanding of the ubiquitination machinery will provide new insights into the regulation of the cell cycle transition, cancer treatment, and the development of anti-cancer drugs.

## 1. Introduction

**Figure 1:**
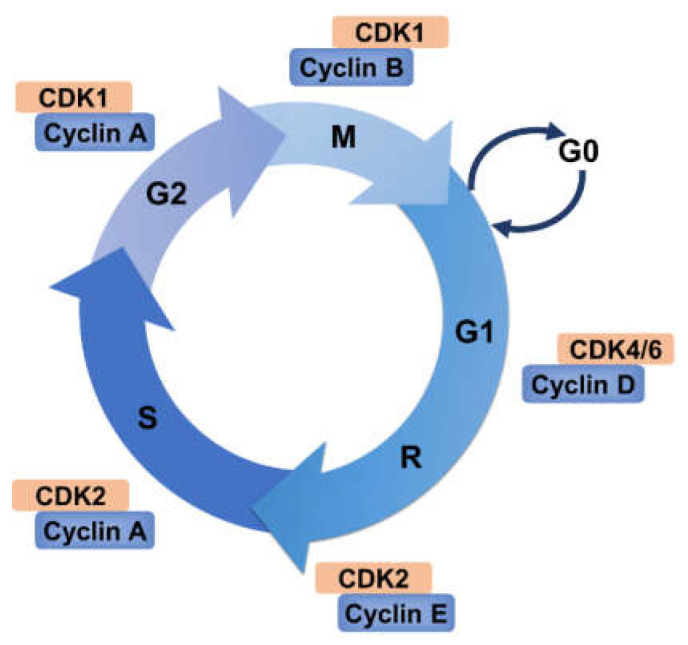
A brief overview of the cell cycle. The normal cell cycle consists of four phases: Gap 1 (G1), DNA synthesis (S), Gap 2 (G2), and mitosis (M). Most nondividing cells exit from the cell cycle and go into quiescent state (G0) but also can re-enter the cell cycle under appropriate stimulation. A cell is committed to entering the cell cycle after passing the restriction point (R). Each phase in cell cycle progression is driven by the activities of cyclin–CDK complexes.

The cell cycle is a ubiquitous, complex, and highly regulated process that is involved in the sequential events during which a cell duplicates its genetic materials, grows, and divides into two daughter cells. Many cellular proteins engage in controlling progression through the four cell cycle phases: G1, S (DNA synthesis), G2, and M (duplication of genetic materials and even distribution). Research during the past several decades has unraveled that the transition of cell cycle phases is mainly driven by phosphorylation of many target proteins via cyclin-dependent kinase (CDK)–cyclin complexes [1,2,3] (Figure 1). CDK activity is closely related to its regulatory subunit cyclins, which are expressed periodically during cell cycle progression [3]. In contrast to most cyclins driving CDK activity, cyclin-dependent kinase inhibitors (CKIs) suppress CDK activity [4]. Thus, close cooperation between this trio is necessary to ensure orderly progression through the cell cycle. In addition to binding cyclins, phosphorylation and dephosphorylation events also govern the CDK activity [5]. CDK7, together with cyclin H and MAT1, forms the CDK-activating kinase (CAK), which allows the T-loop phosphorylation required for activation of CDKs 1,2, 4 and 6, thus driving cell cycle progression [6,7,8,9]. Furthermore, other kinase and phosphatase families also play important roles in the cell cycle, such as Aurora kinase, Polo-like kinase (PLK), Wee1 kinase, Spindle Assembly Checkpoint (SAC) kinase, Never-in-mitosis Aspergillus (NIMA)-related kinases (NEK), checkpoint kinases and CDC25 phosphatase. Aurora kinases (Auroras A, B and C) were initially identified as protein kinases essential for error-free chromosome segregation during mitosis and meiosis. In humans, the most studied of the PLKs is surely PLK1, which directs multiple aspects of the cell cycle process. Van et al. found that PLK1 controls recovery from a G2 DNA damage-induced arrest in mammalian cells [10]. In contrast, PLK1 is dispensable for mitotic entry in unperturbed cell cycles [10]. Of note, Macůrek et al. revealed that PLK1 is activated by aurora A to promote checkpoint recovery [11]. Wee1 is a tyrosine kinase that plays a critical role in the G2–M cell cycle checkpoint arrest for DNA repair before mitosis entry. It directly phosphorylates CDK1 and CDK2 and inhibits their activities [12,13]. The CDC25 (CDC25A, -B and -C) phosphatases are key regulators of normal cell division and the cellular response to DNA damage. The inhibition of Wee1 kinase on CDC2 can be reversed by the human CDC25C protein, which catalyzed the dephosphorylation of CDC2 [13]. SAC is a cell cycle surveillance pathway that inhibits chromosome–segregation in response to unattached kinetochores. BUB1 and BUBR1, the central component of SAC kinases, participate in this correct chromosome-spindle attachment [14], while MPS1 and APC form a feedback loop to allow cells to irreversibly inactivate the checkpoint during anaphase [15]. NEKs, have been identified in most eukaryotes, including humans where eleven genetically distinct proteins named NEK1 to NEK11 are expressed. It is worth noting that NEK2, NEK6, NEK7 and NEK9 contribute to the establishment of the microtubule-based mitotic spindle, whereas NEK1, NEK10 and NEK11 have been implicated in the DNA damage response [16]. Checkpoint kinases CHK1 and CHK2 are serine/threonine kinases which are activated in response to diverse genotoxic insults (reviewed in [17,18]). Cellular responses to DNA damage are mainly regulated by two distinct kinase signaling cascades, the ATR-CHK1 and ATM-CHK2 pathways, which are activated by DNA double-strand breaks (DSBs) and single-stranded DNA respectively (reviewed in [19]). 

The post-translational modification (PTM) of the cell cycle-related proteins is predominantly achieved by two types of protein modifications—phosphorylation and ubiquitination, for qualitative and quantitative control, respectively [20]. Indeed, in recent years, increasing studies have reported that the highly conserved ubiquitin-proteasome system (UPS) plays a critical role in various kinds of cellular processes, including cell cycle control, DNA replication, and DNA damage response [21,22]. Highly specific protein degradation provides direction, order, and proper timing of cell cycle events. Importantly, ubiquitin-mediated proteasome degradation can be reversed by deubiquitylating enzymes (also known as deubiquitinases, DUBs) by removing polyubiquitin chains from substrate proteins. Ubiquitylation and deubiquitylation can regulate the stability, localization, and function of target substrates.

In this review, we focus on and summarize the studies about E3 ubiquitin ligases and DUBs and their functional roles in cell cycle regulation. We illustrate how the UPS regulates cell cycle transition, along with its impact on cancer progression. 

## 2. Cell Cycle and Its Regulation

A decisive characteristic of life is the reproductive capacity of cells, which it does through a collection of highly complex and ordered regulatory process commonly known as the cell cycle [23]. The cell cycle combines DNA replication with chromosomal segregation in an oscillatory manner [24]. In this way, the cell cycle coordinates the precise replication of the genome through specific events to ensure that the duplicated genetic material is distributed equally to each daughter cell. The repetition of this process leads to the exponential proliferation of cells. This process is classically described as interphase and mitosis (M) phase. Most of the cell cycle is in interphase, which encompasses Gap 1 (G1), synthesis (S), and Gap 2 (G2) phases. During the interphase, the cell grows, replicates genetic materials, and repairs DNA damage and replication errors. M phase, a relatively short period, consists of prophase, metaphase, anaphase, and telophase, which completes the equal distribution of genome and cytoplasmic components [1]. Following interphase, most nondividing cells exit the cell cycle at G1 and go into G0 phase (quiescence) [25]. G0 was originally used to describe cells that are not in the cell cycle but with the potential for division [1]. The rate of cell cycling varies with the developmental stage and cell type [26]. In general, the cell cycle is most active during development, as cells in early embryos can proliferate and differentiate to form tissues and organs [27]. The cell cycle involves numerous life processes, and it is closely related to the growth and proliferation of eukaryotic cells, development of organisms, regulation of DNA damage repair [28], and occurrence of diseases [28,29]. 

### 2.1. Cyclin–CDK Complexes Regulation

Studies in the past few decades have revealed that the cell cycle is tightly regulated by complexes containing CDKs and cyclin proteins. CDKs belong to a highly conserved family of serine/threonine protein kinases whose activity relies on a regulatory subunit, a cyclin. The original member of the CDKs family, CDK1 (also known as CDC2), was discovered in genetic screens for *Schizosaccharomyces pombe* and *Saccharomyces cerevisiae* [30]. The proteins of cyclin family which vary with cell cycle progression, are classified by the existence of a CDK-binding domain called the cyclin-box [31,32]. Cyclins were first identified by Tim Hunt in developing sea urchin eggs, and they were synthesized during interphase and then rapidly destroyed at each cleavage division [33]. Up to now, studies showed that fungi contain 6–8 CDKs and 9–15 cyclins, flies and Echinodermata include 11 CDKs and 14 cyclins, and humans have 20 CDKs and 29 cyclins [34]. Many of these 29 cyclins lack known CDK partners, and not all cyclins are involved in cell cycle control, such as cyclin I [35]. 

Different stages of the cell cycle require different cyclins. CDK4 and CDK6 are frequently regarded together as promoters of G1 progression. Initially, mitogenic signaling induces synthesis and assembly of the D-type cyclins, the proper folding of CDK4 and/or CDK6 and their transport to the nucleus [36]. CDK4 or CDK6 is activated after binding to D-type cyclins (D1, D2, and D3). Active complexes of CDK4/6 and D-type cyclins phosphorylate members of the retinoblastoma protein (pRb) family, which includes Rb/p105, p107, and Rb2/p130. Rb protein is a tumor suppressor which plays a key role in the negative regulation of cell cycle and tumor progression. It has been demonstrated that pRb is responsible for preventing S phase entry and cell growth. Phosphorylation of Rb protein leads to their functional inactivation [37]. CDK2 associates with cyclin E during late G1 phase and subsequently binds to cyclin A during early S phase for DNA synthesis and replication [38]. Therefore, both the D- and E-type cyclins and their associated kinases are considered to be pivotal for entry into G1 and G1/S phase transition. As the cell cycle progresses, Cyclin A subsequently interacts with and activates CDK1 in late G2 phase to facilitate the onset of M phase. At last, CDK1 is activated by B-type cyclins, thus triggering chromosome condensation and mitotic entry after nuclear lamina breakdown [30]. 

### 2.2. CKIs Regulation

The activity of each CDK is not only controlled by the availability of its cyclin partner, but it is also negatively regulated by the expression of a specific CDK inhibitor (CKI). CKIs bind to free CDKs or cyclin–CDK complexes to regulate CDK activity. CKIs are classified into two families according to their structures and CDK targets: (1) the CIP/KIP proteins, including p21^CIP1^, p27^KIP1^, and p57^KIP2^, extensively influence the activity of cyclin D-, cyclin E-, and cyclin A-dependent kinase complexes; (2) the INK4 proteins, P16^INK4a^, p15^INK4b^, p18^INK4c^, and p19^INK4d^, specifically target CDK4 and CDK6 [4,39,40]. The lack of these proteins leads to loss of function in growth control, DNA replication, cell apoptosis, and wound repair. Therefore, CKIs are considered as potential targets for cancer treatment.

### 2.3. The Restriction Point and Checkpoints

In fact, Arthur Pardee discovered as early as 1974 that a normal cell needs exogenous mitogenic stimulation only during the first two-thirds of the G1 phase. After undergoing continuous mitogenic stimuli during this period, the cell could decide to continue and complete its cell cycle in the absence of mitogenic signals. This phenomenon suggests the existence of a key point at the mid/late G1 phase, which was termed the “restriction point” (R point) by Pardee [41,42]. Therefore, the R point is the time after which the cell is committed to entering the cell cycle. The R point is considered to be regulated largely by pRb [43].

Furthermore, these regulatory roles of cyclin–CDK complexes allow for checkpoints during the cell cycle, which are inhibitory pathways. Cell cycle checkpoints have been defined as “biochemical pathways that ensure dependence of one process upon another process that is otherwise biochemically unrelated.” Checkpoints can control cell cycle progression and ensure high-fidelity completion of crucial events such as DNA replication and chromosome segregation [44]. Regulatory checkpoints include the DNA damage and DNA replication checkpoints and spindle assembly checkpoint. The DNA damage checkpoint is responsible for detecting damaged DNA and generating arrest in G1, S, and G2 phase of the cell cycle. The G1 arrest provides time for DNA repair before replication, while the block in G2 allows repair before chromosome separation in M phase. Cells can also be arrested in S phase, slowing down DNA synthesis [1]. The DNA replication checkpoint ensures that mitosis does not take place until DNA replication is complete, otherwise cells face the risk of chromosome mutation [45]. The spindle assembly checkpoint is one of the critical mechanisms to ensure the correct and even distribution of genetic material into two daughter cells. It monitors the binding of chromosomes and spindle microtubules as well as the combination of chromosomes and spindle microtubules [46]. Recently, a growing body of evidences suggested that checkpoint dysfunctions result in genomic instability which have been related to cancer.

## 3. The UPS Molecular Machinery

**Figure 2:**
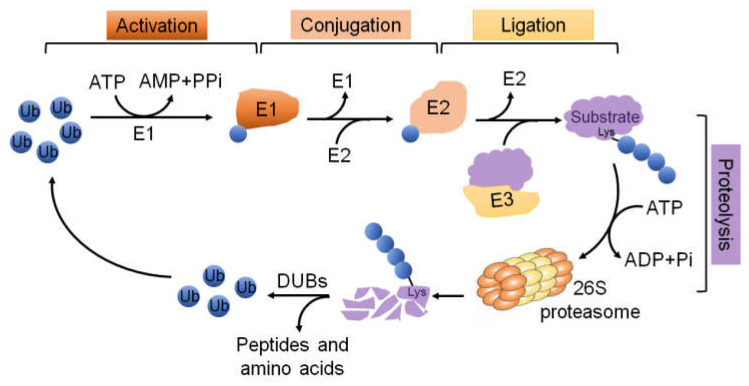
Overview of the ubiquitin-proteasome system. Ubiquitination is a multistep enzyme cascade. The first step is that free ubiquitin (Ub) is activated by an E1 ubiquitin-activating enzyme in an ATP-dependent manner. Then, the activated ubiquitin is transferred to an E2 ubiquitin-conjugating enzyme and is attached to a substrate protein through the actions of an E3 ubiquitin-ligase enzyme, which identified substrate specifically. Finally, the ubiquitinated substrate protein is subsequently recognized by the 26S proteasome and degraded into small peptides and amino acids. Deubiquitinating enzymes (DUBs) reverse the ubiquitination process through removing polyubiquitin chains from proteins to maintain intracellular ubiquitin levels.

**Figure 3:**
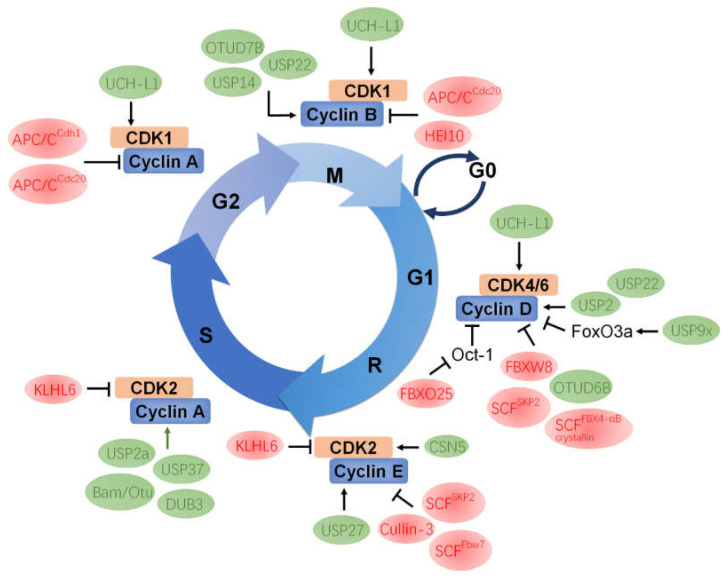
E3 ubiquitin ligases and DUBs regulate cell cycle by targeting cyclins and CDKs. E3 ubiquitin ligases (red ovals) trigger the ubiquitylation and proteolysis of cyclins and CDKs at specific stages of the cell cycle. Most deubiquitinating enzymes (green ovals) deubiquitinates and stabilizes cyclins and CDKs, promoting cell cycle progression. However, OTUD6B is likely to participate in the downregulation of cyclin D2 through an intermediate, but the mechanism is not yet clear. UCH-L1 does not control the cell cycle by stabilizing substrate proteins, but physically interacts with CDK1, CDK4, and CDK5 and enhances CDKs activity. Refer to the main text for details.

The expression levels of cyclins during cell division are periodic. This is the result of a constant synthetic rate coupled with specific proteolysis, which is executed by the UPS. In the cell cycle, ubiquitination plays a central role in cell cycle transitions and checkpoints by establishing the strict temporal control of proteins such as cyclins, CDKs, CKIs, other kinases and phosphatases [21]. The UPS is one of the protein degradation pathways in eukaryotic cells, and most proteins rely on this system for degradation. The UPS consists of ubiquitin, E1 ubiquitin-activating enzymes, E2 ubiquitin-conjugating enzymes, E3 ubiquitin–ligase enzymes, deubiquitinating enzymes (DUBs), and 26S proteasome [47]. Degradation of target proteins requires a multistep reaction (Figure 2). The first step is to covalently conjugate a single ubiquitin or several ubiquitin molecules to the substrate protein mediated by E1, E2, and E3 enzymes. Then, the 26S proteasome selectively degrades based on different ubiquitin connection types [48]. Ubiquitination is a reversible modification, and DUBs can remove the ubiquitin chain from the substrate protein [49]. For instance, K11- or K48-polyubiquitin chains mediate proteasome degradation [50], whereas K63-polyubiquitin conjugates are involved in regulating cellular signal transduction [51]. In addition, mono-ubiquitination has been shown to be implicated in the endocytosis of plasma membrane receptors [52,53]. Many studies have demonstrated that ubiquitination can participate in cell cycle regulation, cell apoptosis and autophagy, ER stress, and protein localization and activity regulation. Of relevance, the regulation of the cell cycle is one of the cellular processes which have to be loyally coordinated and regulated by ubiquitination [54] (Figure 3). For example, the principal ubiquitin ligase families involved in cell cycle regulation are the SCF and anaphase promoting complex/cyclosome (APC/C) complexes [3,20,21]. The human APC/C consists of 19 core subunits and is an evolutionarily conserved multi-subunit cullin-RING E3 ubiquitin ligase which is primarily known for its ability to trigger the transition from metaphase to anaphase during mitosis [55]. APC/C is controlled by two major activators, Cdc20 in metaphase and Cdh1 in telophase. Either one can activate APC/C to target the degradation of specific substrates, including cyclins, mitotic kinases (such as Polo-like kinases, NIMA-related kinases, and Aurora kinases), spindle assembly factors, and DNA replication proteins during different time of the cell cycle [3,56,57,58]. Binding of Cdc20 or Cdh1 to the APC/C induces a conformational change to the active forms, APC/C^Cdc20^ and APC/C^Cdh1^, respectively. APC/C^Cdc20^ exists in the onset of mitosis until the beginning of anaphase. In early mitosis, Cdc20 becomes active and associates with phosphorylated APC/C by CDK1, resulting in the degradation of prometaphase substrates, such as cyclin A [56,59,60]. Once all chromosomes achieve proper positions in metaphase, APC/C^Cdc20^ degrades Securin and cyclin B to facilitate cellular transition to anaphase. Securin is an anaphase inhibitor and is essential for chromosomal separation [61]. Therefore, APC/C^Cdc20^ mediates the separation of sister chromatids and is the target of regulatory mechanisms whose role is to ensure that daughter cells inherit complete copies of the genome [62], whereas APC/C^Cdh1^ is present in the late mitosis to late G1, promoting efficient mitotic exit and maintenance of the G1 state. Similarly, APC/C^Cdh1^ also mediates the degradation of mitotic cyclins [63]. Furthermore, APC/C^Cdh1^ induces the degradation of SKP2, which, in turn, targets for the degradation of the CKIs such as p27, p21, and p57 [64,65].

APC/C is mainly active in mitosis and G1 phase of the cell cycle and acts as the key antagonist of mitotic CDKs [62], while SCF complexes is always active, executing a variety of functions at most phases of the cell cycle and beyond. The SCF complex is comprised of the invariable components SKP1, CUL1, and RBX1 as well as one variable component, known as an F-box protein, that binds to SKP1 through its F-box motif and is responsible for substrate recognition [66]. To date, about 70 F-box proteins have been identified in humans, and these proteins are divided into three categories: those with WD40 repeats (FBXW), leucine-rich repeats (FBXL), or other domains (FBXO) [67]. Some of them, e.g., FBXW8 [68], FBXO25 [69], Fbw7 [70], and SKP2 [71,72,73,74], can directly regulate cyclins or CKIs. More importantly, not only APC/C and SCF cooperate in the regulation of common substrates, but also their activities are regulated by each other. As noted above, APC/C^Cdh1^ ubiquitinates SKP2 during G1 phase, allowing for the post-mitotic down-modulation of SCF [64]. Furthermore, Emi1 is a protein able to inhibit the activity of APC/C in S and G2 phases [75]. Emi1 is phosphorylated by Cdc2 and is subsequently recognized by the SCF^β-TRCP^ ubiquitin ligase and destroyed in prophase, thus promoting APC/C activation [76].

## 4. The Functional Roles of UPS in Cyclins

Ubiquitin-dependent degradation plays an essential role in many important cellular processes such as cell cycle regulation and signal transduction. In the cell cycle, ubiquitin-dependent proteolysis provides a rapid control for modulating the protein expression levels of cyclins. The accumulation or degradation of cyclins will lead to different cell fates (Table 1).

### 4.1. Cyclin A

Cyclin A plays a fundamental role in regulating cell cycle events because it can activate CDK2 and CDK1, and has functions in both S and M phase [77]. A study has found that human cyclin A is relatively stable during S and G2 phase, but it is rapidly degraded when cells enter metaphase of mitosis [78]. There are two types of cyclin A (cyclin A1 and cyclin A2) in *Xenopus* [79,80], mice [81], and humans [82], both of which can form complexes with either CDK1 or CDK2. Cyclin A1 is only expressed in meiosis and very early embryos, whereas cyclin A2 exists in proliferating somatic cells [78]. A previous study has shown that the APC/C is an evolutionarily conserved multi-subunit E3 ubiquitin ligase composed of 14 proteins, which can target cyclin A for ubiquitin-mediated degradation. The degradation of cyclin A by Cdc20 or Cdh1-activated APC/C is necessary for the onset of anaphase and exit from mitosis, in contrast to nondegradable cyclin A arrests cells in anaphase [55,78]. However, the ubiquitination of cyclin A can be reversed by USP37. USP37 binds, deubiquitinates, and stabilizes cyclin A, thereby regulating the G1/S transition [83]. In the identification of other DUBs of cyclin A, it was found that USP2a can bind to and negatively stabilize cyclin A, resulting in enhanced oncogenic characteristics in bladder cancer cells [84]. Subsequent research in lung cancer cells has identified DUB3 (also termed USP17) as a new regulator of cyclin A. DUB3 binds to cyclin A and promotes its deubiquitination and stabilization. Compared with the control cells, the percentage of S phase cells was significantly decreased in DUB3 knockdown A549 cells, whereas this effect can be rescued by overexpression of cyclin A in DUB3 depletion cells. These results suggest that the DUB3–cyclin A axis plays a key role in G1/S transition during cell cycle progression and at least partially explain the mechanism of DUB3 which promotes the proliferation of NSCLC cells, providing a promising target for NSCLC treatment [85]. 

In *Drosophila*, it has been demonstrated that fate determination of germ-line stem cells (GSCs) (such as differentiation or regeneration) is tightly controlled by a specific cell cycle program [86]. Previous studies have revealed that mutation or dysregulated expression of cyclins, such as cyclin A and cyclin E, leads to defects of GSC maintenance, differentiation, and/or an abnormal cell number of cyst [87,88,89]. Thus, correct expression of cyclins is important for GSC fate determination and the normal development. A recent study in *Drosophila* uncovered that Bag of marbles (Bam) can form a deubiquitinase complex with Otu to deubiquitinate and stabilize cyclin A, thereby balancing GSC self-renewal and differentiation [90]. 

### 4.2. Cyclin B

On the one hand, cyclin B is the key regulatory protein controlling mitosis in all eukaryotes and forms a complex with CDK1 to initiate the mitotic program through phosphorylation of selected proteins. On the other hand, destruction of cyclin B due to ubiquitin-mediated proteolysis is necessary for mitotic exit [106]. Therefore, the abundance of cyclin B is critical to cell cycle progression. Interestingly, the APC/C protein complex can target both cyclin A and cyclin B. In the late 20th century, scientists proved that degradation of B-type cyclins depends on ubiquitination which is mediated by the activity of the APC/C^Cdc20^ complex [91,107]. Later in 2003, human enhancer of invasion 10 (HEI10) was first identified as a RING-finger family ubiquitin ligase that regulates cell cycle by interacting with cyclin B1 and promoting its degradation, thereby specifically functioning in progression through G2/M [92]. 

Researchers have also explored the mechanism of deubiquitination of cyclin B. Studies have found that the deubiquitinating enzyme USP14 seems to be closely related to cyclin B. USP14, belonging to the USP family, which binds reversibly to the 26S proteasome [108], limits proteasomal degradation of ubiquitinated proteins [109,110]. Lee et al. reported that USP14 shows a dramatic preference for ubiquitin–cyclin B conjugates that carry more than one ubiquitin modification or chain [110]. A recent study further verified that USP14 can interact with cyclin B1, promoting its deubiquitination and stabilization. Knocking down USP14 with siRNAs significantly inhibited the proliferation and migration of breast cancer cells. Thus, these results provide a solid theoretical basis for the development of anti-cancer drugs targeting USP14 [97]. Cyclin B is also regulated by multiple DUBs. USP22 can interact with cyclin B1, promoting its deubiquitination and stabilization. Interestingly, USP22 is also phosphorylated by CDK1, and this phosphorylation enhances its activity in deubiquitinating cyclin B1. Compared with the wild-type control, knockdown of *usp22* gene in colon cancer HCT116 cells or in mouse embryonic fibroblast cells leads to decreased cell proliferation [98]. Thus, USP22 is involved in cell proliferation possibly through promoting cyclin B1 stabilization. In addition, another study has also found that ovarian tumor domain-containing 7B (OTUD7B) is a cell cycle-regulated deubiquitinase that antagonizes the degradation of APC/C substrates. OTUD7B can deubiquitinate cyclin B by specifically removing the K11-linked ubiquitin chain from cyclin B, thereby protecting cyclin B from 26S proteasome-mediated degradation. Further observations unraveled that OTUD7B contributes to mitotic exit and that its function is crucial for cells to properly progress through the cell cycle and proliferate [99]. 

### 4.3. Cyclin D

It is well known that D-type cyclin regulates G1 progression and is overexpressed in various human cancers [111,112]. Therefore, cyclin D is considered as one of the potential therapeutic targets for cancer [113]. SKP1–CUL1 (Cdc53 in yeast)–F-box-protein (SCF) is one of the important E3 ubiquitin ligases that regulate D-type cyclins, and its substrate specificity is determined largely by F-box protein (FBP) [67]. As early as 1998, Zhang’s team identified that human Cullin 1 (CUL-1) associates with SKP1/SKP2 to form the SCF^SKP2^ complex. This complex may play function as a conserved E3 ubiquitin ligase, which is similar to yeast SKP1–CDC53–F-box protein complex. They proposed that this complex is likely to target cyclin D and p21 for ubiquitin-dependent degradation, although this view needs further biochemical analysis to support [71]. Okabe et al. found that FBXW8, the F-box protein, could specifically associate with cyclin D1 in a Thr286 phosphorylation-dependent manner and regulate its stability through the UPS pathway. Interestingly, depletion of FBXW8 leads to a significant accumulation of cyclin D1, while preventing cyclin D1 from entering the nucleus and resulting in a slower rate of cell proliferation [68]. A recent study has shown that another F-box protein FBXO25 also regulates cyclin D1, but this regulation is not achieved through the direct interaction between FBXO25 and cyclin D1. FBXO25 directly interacts with the cyclin D1 repressor Oct-1 and downregulates its expression [69]. It is well known that Oct-1 as transcription factor binds to the promoter of cyclin D1 and leads to its inactivation [93]. Therefore, the decrease of Oct-1 is conducive to stabilize cyclin D1, thereby promoting tumor growth and metastasis [69]. In another study, SCF^FBX4-αB crystallin^ complex was identified as an E3 ubiquitin ligase of cyclin D1, promoting ubiquitin-dependent degradation of Thr286 phosphorylated cyclin D1. Knockdown of SCF^FBX4-αB crystallin^ ligase can increase the accumulation of cyclin D and accelerate G1 phase progression [94]. 

Ubiquitination of proteins is a dynamic process which can be reversed by removal of ubiquitin by DUBs. Thus, cyclin D1 is also regulated through a specific DUB. In 2009, a novel regulator of cyclin D1 was described by Shan et al., who identified USP2 as a deubiquitinase responsible for cyclin D1 stabilization. The accumulation of cyclin D1 accelerates the G1 to S phase progression. Of note, restraint of USP2 function induced growth suppression only in the cancer cells addicted to cyclin D1 expression, whereas there was no major effect on cell growth of normal human fibroblasts [100]. This different effect of USP2 in normal and cancer cells suggests that USP2 may be used as a new drug target to block its activity and reduce the proliferation of cancer cells in a safe and non-toxic way. In 2017, Magiera et al. found that lithocholic acid hydroxyamide (LCAHA) destabilizes cyclin D1 and induces G0/G1 arrest by inhibiting activity of USP2 deubiquitinase [114]. In addition, three distinct classes of small-molecule inhibitors of USP2 have been reported: NSC 632839 [115], chalcones [116], and the compound ML364 [117]. It has been reported that the cell cycle arrest induced by FoxO forkhead transcription factors (such as FoxO4, FoxO3a, and FoxO1a) involves the reduced protein expression of D-type cyclins [102]. Thus, these transcription factors are negative regulators of D-type cyclins. Zheng et al. found that USP9x can interact with FoxO3a and reduce the ubiquitination level of FoxO3a and stabilize it. At the same time, FoxO3a is also regulated by prolyl hydroxylase EgIN2. Prolyl-hydroxylated FoxO3a prevents the binding of USP9x, thereby promoting the proteasomal degradation of FoxO3a and the accumulation of cyclin D1 [101]. Another study provided evidence that OTUD6B probably participates in cell cycle progression of B lymphocytes by regulating cyclin D2. Meanwhile, the deubiquitinating activity of OTUD6B on cyclin D2 was not observed [103]. Therefore, OTUD6B is likely to be involved in the regulation of cyclin D2 through an intermediate, but it is not yet clear. Apart from that, a recent study has revealed that USP22 regulates cell cycle by directly deubiquitylating cyclin D1, thus protecting cyclin D1 from proteasome-mediated degradation. Loss of USP22 in cancer cells results in defective G1/S cell cycle transition, whereas ectopic cyclin D1 protein partially rescues the aberrant cell proliferation phenotype. It suggests that USP22 plays a significant role in cell cycle progression via stabilization of cyclin D1 [104].

### 4.4. Cyclin E

Cyclin E binds and activates CDK2 and promotes the G1/S transition of the cell cycle. The amount of cyclin E is tightly regulated by ubiquitin-mediated proteolysis. Cyclin E accumulation can lead to accelerated S phase entry [118], genetic instability [119], and tumorigenesis [120]. Cullin-3 (Cul-3) is a member of the cullin family of E3 ubiquitin–protein ligases. A study has shown that Cul-3 can bind to free cyclin E but not to the cyclin E/CDK2 complex in mammalian cells, and it specifically increased ubiquitination of cyclin E. Deletion of the *Cul-3* gene in mice caused increased accumulation of cyclin E. In the extraembryonic ectoderm and ectoplacental cone of the *Cul-3^−/−^* embryos, there was a great increase of S phase cells [95]. A recent study further proved that Cul-3 regulates cyclin E1 protein abundance via a degron located within the N-terminal region of cyclin E. However, cyclin E2 lacks the Cul-3 degron and is not targeted for degradation by Cul-3 [121]. Cyclin E is also regulated by multiple E3 ubiquitin ligases. Koepp et al. identified that the E3 ubiquitin ligase complex SCF^Fbw7^ is responsible for cyclin E ubiquitination and demonstrated that it is functionally conserved in yeast, flies, and mammals. Fbw7, as the FBP component of SCF protein complex, can specifically associate with cyclin E and promote its ubiquitination. Deletion of Fbw7 results in the accumulation and stabilization of endogenous cyclin E in human and *Drosophila melanogaster* cells [70]. Thus, Fbw7 is a negative regulator of cyclin E and a potential tumor suppressor. In another study in the same year, it was also proved that another important F-box protein SKP2 can negatively regulate the stability of cyclin E [96]. It has been previously shown that phosphorylation of threonine 380 (Thr380) at the carboxy terminus of cyclin E provides a signal for the ubiquitin-dependent proteolysis of cyclin E [122,123]. They identified that SKP2 specifically binds to cyclin E, which is phosphorylated at Thr380 and mediates its ubiquitin-dependent degradation, thus contributing to the control of S phase progression [96]. 

Cyclin E abundance is also regulated by a specific DUB. In 2018, USP27 was identified as the DUB of cyclin E [105]. Both the C-terminal UCH domain of USP27 and the N terminus of Cyclin E are required for this interaction. USP27 can reduce the ubiquitination level of cyclin E, thereby promoting cyclin E stabilization. Furthermore, knockdown of USP27 suppresses cell cycle progression by increasing the percentage of cells in G0/G1 phase, which can be reversed by the recovery of cyclin E expression [105]. 

## 5. Mutual Regulation between CDK and UPS Components

Protein phosphorylation is a key regulatory mechanism for cell cycle control in eukaryotes. From yeast to humans, cell cycle progression and cell division require the activation of CDKs. CDKs are activated by associating with a cyclin, then initiating and coordinating these processes by orderly phosphorylation of their targets. Therefore, CDK is not only regulated by ubiquitination and/or deubiquitination, but also exerts kinase activity to phosphorylate substrate proteins. In this section, we pay attention to the regulation of CDKs by UPS and discuss the phosphorylation regulation of some deubiquitinases and E3 ligases by CDKs (Table 2). 

### 5.1. CDK1

The onset of mitosis requires increased association activity of CDK1 with cyclin A and cyclin B. The CDK1/cyclin B complex is the dominant regulator of the G2/M transition and has maximal activity during metaphase. Yang et al. reported that CDK1 phosphorylates the histone H2A deubiquitinase Ubp-M at serine 552 (S552P), and, importantly, this phosphorylation is required for cell cycle progression. Ubp-M (S552P) destroys Ubp-M interaction with chromosome maintenance protein 1 (CRM1), allowing Ubp-M to be retained in the nucleus and deubiquitinate nucleosomal uH2A, which is required for chromosome condensation and cell cycle progression [124]. In addition, Ubiquitin C-terminal Hydrolase L1 (UCH-L1/PGP9.5) is a deubiquitinating enzyme which hydrolyzes C-terminal esters and amides of ubiquitin [125]. Kabuta et al. proved that UCH-L1 physically interacts with CDK1, CDK4, and CDK5 and enhances CDKs activity. To investigate the mechanism how UCH-L1 enhances the kinase activity of CDK, they used UCH-L1^I93M^ and UCH-L1^C90S^ mutants in cell-based kinase assays. Their observations indicated that the enhancement of CDK activities by UCH-L1 is independent of the known ubiquitin-related functions of UCH-L1 but correlates with interaction levels between UCH-L1 and CDKs. Besides that, overexpression of UCH-L1 enhances cell proliferation, and knockdown of UCH-L1 reduces tumor growth and CDK kinase activity [126]. The findings provide us with a promising strategy by targeting UCH-L1 to treat cancer.

### 5.2. CDK2

CDK2 plays a pivotal part in cell cycle regulation and is involved in a range of biological processes. It is also controlled by both ubiquitination and deubiquitination. The COP9 signalosome (CSN) is a DUB of the JAMM family composed of eight subunits (CSN1-8) and well-conserved in all eukaryotes from yeast to humans [127,128]. CSN5 is the only CSN subunit possessing the JAMM motif, and it was assumed that CSN5 has a deubiquitylating activity on its own [129]. Yoshida et al. found that CSN5 specifically interacts with CDK2 and positively regulates CDK2 stabilization. Interestingly, the expression level of cyclin E increased in accordance with CDK2’s upregulation, suggesting that CSN5 also regulates the level of cyclin E through CDK2. In addition, G1 progression of the cell cycle is markedly hampered in CSN5-depleted cells [127]. In a later study, it was verified that Kelch-like protein 6 (KLHL6), as an E3 ubiquitin ligase, plays an important role in acute myeloid leukemia (AML) by regulating the expression of CDK2. KLHL6 is the E3 ligase that binds to CDK2 and mediates its degradation [130]. 

### 5.3. CDK4/6

CDK4/6 are activated by the D-type cyclins, thereby phosphorylating pRb to promote G1/S transition. A recent study revealed that the CDK4/6–DUB3–SNAIL1 axis functions as an important regulatory mechanism of breast cancer metastasis [131]. SNAIL1 is deemed to be a key factor in the aggressive expression of tumors for its critical role in the epithelial–mesenchymal transition (EMT) pathway associated with tumor metastasis [132]. Liu et al. identified DUB3 as a new target of CDK4/6, and CDK4/6 phosphorylates DUB3 at serine 41. As a linker between CDK4/6 and SNAIL1, DUB3 activation by CDK4/6 is essential to deubiquitinate SNAIL1. As a bona fide SNAIL1 deubiquitinase, DUB3 interacts with SNAIL1, and then specifically removes the K48-linked ubiquitin chain to stabilize SNAIL1. Furthermore, knockdown of either DUB3 or CDK4 or CDK6 markedly decreased the expression level of SNAIL1, thus regulating cell migration and cancer metastasis [131]. 

## 6. Ubiquitination and Deubiquitination Involved in CKIs

**Figure 4:**
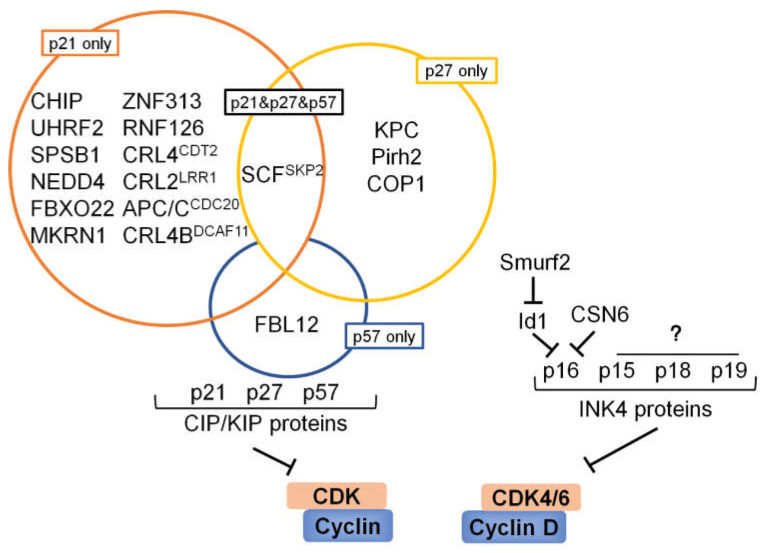
Summary of E3 ubiquitin ligases involved in the regulation of CKIs. Summary of E3 ligases that regulate cell cycle progression by targeting p21, p27, p57, or p16. p21, p27, and p57 are all controlled by SCF^SKP2^. Currently, the identification of the E3 ligase of the INK4 protein family is still insufficient.

The kinase activity of cyclin–CDK complexes is tightly regulated by a plethora of CKIs which serve as brakes to halt cell cycle progression under adverse conditions. Similar to cyclins and CDKs, the expression level of CKIs is also balanced by ubiquitination and deubiquitination (Table 3 and Figure 4). 

### 6.1. p53 and Its Downstream Target p21

As one of the most well-known and important tumor suppressors, p53 has received widespread attention and study. One central role of the tumor suppressor p53 is to arrest the cell cycle, as p53 indirectly downregulates the expression of many genes which are essential for progression through the cell division cycle [133]. Notably, many studies cemented that p53 is commonly mutated in most tumor types, with conservative estimates indicating a mutation rate of 50% across all tumors [134,135,136,137]. In normal cells, p53 is maintained at very low, often at undetectable levels. Murine double minute 2 (MDM2) is the main negative regulator of p53 activity and stability. Several studies have revealed that diverse signals regulate p53 levels by regulating the interaction between MDM2 and p53 [138,139]. First, MDM2 binds the transcriptional activation domain of wild-type and mutant p53, and blocks its ability to regulate target genes [140,141]. Second, MDM2 can act as an E3 ubiquitin ligase and interact with p53 to promote its ubiquitination and 26S proteasome-mediated degradation [142]. Importantly, p53 activates the expression of *mdm2* gene in an autoregulatory feedback loop which aims to maintain low cellular p53 levels in the absence of stress [140,143]. The inhibitory effect of MDM2 on p53 can be reversed by E3 ligase of MDM2, such as PCAF [144], SCF^β-TRCP^ [145], XIAP [146], RFP2 [147], NAT10 [148], and MARCH7 [149]. These E3 ubiquitin ligases increase the stability of p53 by reducing p53 ubiquitination through ubiquitination and degradation of MDM2. In contrast, several deubiquitinating enzymes, such as HAUSP (also known as USP7) [150], USP2a [151], and USP15 [152], are able to stabilize MDM2 by removing its polyubiquitin chains. Intriguingly, HAUSP was initially identified as a deubiquitinase of p53. HAUSP interacts with p53 and removes the ubiquitin from p53, resulting in p53 stabilization. It has been found that the overexpression of HAUSP stabilizes p53 even in the presence of excess MDM2, and the downregulation of HAUSP expression makes the endogenous p53 unstable [153]. Compared with p53, the binding between MDM2 and HAUSP is much stronger [154,155]. Thus, it has also been observed that the disruption of the HAUSP gene stabilizes p53. With the in-depth development of research, more and more ubiquitin ligases that regulate p53 have been discovered, including Pirh2 [156], ICP0 [157], COP1 [158], Topors [159], CHIP [160], ARF-BP1 [161], TRIM24 [162], HRD1 [163], MKRN1 [164], WWP1 [165], TRIM39 [166], TRAF7 [167], Cullin4B [168], TRIM71 [169], RING1 [170], FBW7α [171], and TRIM69 [172]. All of these ubiquitin ligases could target p53 for proteasomal degradation. Great progress has been made in identifying the regulation of p53 ubiquitination; in addition, diverse DUBs that positively regulate p53 stability have also been discovered. HAUSP is the first identified deubiquitinating enzyme for p53, and it is considered to have a dual role in the p53-MDM2 pathway because it deubiquitinates both p53 and MDM2. In a subsequent study, USP10 was also identified as a p53 deubiquitinase which can interact with and stabilize p53. In unstressed cells, USP10 localizes in the cytoplasm and regulates p53 homeostasis. Following DNA damage, a fraction of USP10 translocates to the nucleus and contributes to p53 activation. Moreover, USP10, through its regulation of p53, appears to play an important role in inhibiting cancer cell growth [173]. In addition, Sun et al. revealed that Otub1 interacts with DNA-binding domain of p53 via its N and C termini and positively regulates stability of p53. Moreover, the DUB activity of Otub1 on p53 may depend on its Asp88. Overexpression of Otub1 drastically induces p53-dependent apoptosis and inhibition of cell proliferation [174]. Besides that, a series of studies has demonstrated that USP29 [175], USP42 [176], USP11 [177], USP9x [178], USP24 [179,180], and Ataxin-3 [181] can participate in p53-mediated multiple functions through deubiquitination and stabilization of p53.

p21^CIP1/WAF1^ (p21 herein after) is a potent, tight-binding inhibitor of CDKs and can suppress the phosphorylation of Rb by cyclin A–CDK2, cyclin E–CDK2, cyclin D1–CDK4, and cyclin D2–CDK4 complexes, resulting in G1 cell cycle arrest [182]. Thus, *p21*^−/−^ cells show an impaired G1 checkpoint and do not arrest upon DNA damage and other insults [183,184]. Moreover, *p21* gene is one of the most important downstream targets of *p53* gene and a p53-binding site was identified in 2.4 kb upstream of *p21* coding sequences. Ever since the initial identification of p21 as a direct p53 transcriptional target capable of inducing cell cycle arrest, this protein has been considered an important mediator of p53-dependent tumor suppression [185]. Therefore, both p21 and p53 are negative regulators of the cell cycle, and their stability is crucial for cell cycle progression. We previously discussed that p53 is the target of multifarious E3 ubiquitin ligases and DUBs. Much of the regulation of p21 levels is achieved by transcriptional control mechanisms. However, it appears likely that post-transcriptional processes may also regulate p21 levels [72]. Three different E3 ubiquitin ligases have been identified in targeting p21 degradation: SCF^SKP2^ [71,72,73], APC/C^CDC20^ [186], and CRL4^CDT2^ [187,188,189]. In subsequent research, MKRN1 was reported as an E3 ubiquitin ligase for both p53 and p21, and ablation of MKRN1 induces cell cycle arrest by stabilizing p53 and p21 [164]. In 2010, Starostina et al. demonstrated that the CRL2^LRR1^ complex also targets p21 for degradation in *C. elegans* to promote cell cycle progression in germ cells. However, the degradation of p21 mediated by human CRL2^LRR1^ complex does not appreciably affect cell cycle progression [190]. Up to date, ZNF313 [191], RNF126 [192], CHIP [193], CRL4B^DCAF11^ [194], UHRF2 [195], SPSB1 [196], NEDD4 [197], and FBXO22 [198] have also been added to the growing list of E3 ubiquitin ligases that control the abundance of p21 protein. Compared with the ubiquitination studies of p21, the identified deubiquitinases targeting p21 are much less. Until 2018, Deng et al. identified USP11 as the first deubiquitylase that directly reverses p21 polyubiquitylation and stabilizes the p21 protein. Loss of USP11 causes the destabilization of p21 and induces the G1/S transition in unperturbed cells. Furthermore, DNA damage-mediated p21 accumulation is completely abolished in cells depleted of USP11, which leads to abrogation of the G2 checkpoint and induction of apoptosis [199].

Although the rapid degradation of p53 and p21 are largely achieved through the ubiquitin proteasome pathway, both p53 and p21 are regulated by a ubiquitin-independent process mediated by 20S proteasome [200,201]. In a series of studies, Asher et al. demonstrated that NAD(P)H quinone oxidoreductase 1 (NQO1) could stabilize p53 and revealed that (NQO1) is associated with the 20S proteasomes and interacts with p53 in an NADH-dependent manner to protect p53 from 20S proteasomal degradation [200,202,203]. Another study has shown that proteasome activator REG-γ (also known as PA28, PSME3, and Ki antigen), which binds and activates 20S proteasome [204], plays an important role in regulating p21. They suggested that REGγ directly mediates degradation of free p21 in an ATP- and ubiquitin-independent manner. Overexpression of REGγ reduced p21 level, while depletion of REGγ enhanced the p21 level in multiple cell lines. In addition, depletion of REGγ in a thyroid carcinoma cell line results in cell cycle and proliferative alterations [201]. In conclusion, the stability regulation of p53 and p21 is a very complicated process and is affected by many factors.

### 6.2. p27

The p27 gene is located on chromosome 12p13. p27 protein binds to cyclin (A, B, D, and E)–CDK (1, 2, 4, and 6) complexes and leads to the failure of Rb phosphorylation, which ultimately results in standstill of G1/S transition [205]. Therefore, degradation of mammalian p27 is required for the cellular transition from quiescence to the proliferative state. Previous studies have demonstrated that nuclear SCF^SKP2^ E3 ligase specifically recognizes p27 in a phosphorylation-dependent manner that is characteristic of an F-box-protein–substrate interaction, thus promoting ubiquitin-mediated degradation [74]. Most importantly, phosphorylation of p27 at Thr187 was essential for its degradation [206]. Complete knockdown of SKP2 partially prevented p27 degradation. Furthermore, SKP2 is important in the progression from quiescence to S phase and that the ability of SKP2 to promote p27 degradation is a key aspect of its S-phase-inducing function [207]. However, proteolysis of p27 at the G0/G1 transition proceeds normally in *skp2*^-/-^ cells [208]. Kamura et al. proposed that the existence of a SKP2-independent pathway for the degradation of p27 at G1 phase. They described that the cytoplasmic ubiquitin ligase KPC consisting of KPC1 and KPC2 interacts with and ubiquitinates p27. Moreover, the nuclear export of p27 by CRM1 seems to be necessary for KPC-mediated proteolysis. Overexpression of KPC promoted the degradation of p27, whereas a dominant-negative mutant of KPC1 delayed p27 degradation, and knockdown of KPC1 by RNA interference also inhibited p27 degradation. Further exploration suggested that KPC controls not only the progression from G0 to S phase but also the growth of continuously cycling cells during long-term culture [209]. Subsequently, a study has identified that a RING-H2-type ubiquitin ligase Pirh2 interacts with p27 and facilitates its proteasomal degradation. Depletion of Pirh2 induced accumulation of p27 in both the nucleus and cytoplasm and resulted in an inhibition of cell cycle progression at G1/S transition in a p53-independent manner [210]. It has been reported that COP1 also enhances ubiquitin-mediated degradation of p27 to promote cancer growth [211]. Although CSN is involved in regulating various biological processes, individual subunits of COP9 have been suggested to modulate different physiological functions [212]. Different from CSN5 as mentioned above, subunit 6 of the COP9 signalosome complex (CSN6) usually collaborates with other E3 ligase to regulate target proteins, thereby governing cancer development. One study demonstrated that the CSN6–COP1 axis plays a critical role in controlling the stability of p27 [213]. Depletion of CSN6 results in reduction of COP1 and concurrent elevation of p27. Mechanistic studies showed that CSN6 interacts with p27 and enhances ubiquitin-mediated degradation of p27. CSN6-mediated p27 degradation depends on the nuclear export of p27, which is regulated through COP1 nuclear exporting signal [213]. A previous study has reported that CSN6 associates with COP1 and positively regulates its stability through inhibition of ubiquitin-mediated COP1 proteasomal degradation [214]. Therefore, p27, COP1, and CSN6 possibly form a complex, and CSN6 increases the degradation of p27 by stabilizing COP1. However, it seems that USP37 is the only deubiquitinase directly related to p27. USP37 formed a complex with p27, promoted its deubiquitination and stabilization, and blocked cell proliferation [215]. 

### 6.3. p57

The cyclin/CDK inhibitor p57^Kip2^ is the least studied member of the Cip/Kip family [216]. As one of the most important E3 ligases in cell cycle regulation, the SCF^SKP2^ complex targets multiple substrate proteins including p57 for ubiquitylation and proteolysis [217]. Then, Chiba’s team identified that FBL12, a new F-box protein induced by TGF-β1, could directly ubiquitinate p57 and lead to its degradation. Impairment of FBL12 and overexpression of p57 enhanced osteoblast cell differentiation, indicating the importance of p57 degradation in the TGF-β1-mediated inhibition of osteoblast cell differentiation [218]. Subsequently, it was also proved that p57 can be negatively regulated by CSN6 [219]. CSN6 negatively regulates p57 stability via increased ubiquitination levels of p57, thereby reducing p57′s steady-state expression. Further mechanistic studies have indicated that CSN6 associates with p57 and SKP2, which, in turn, facilitates SKP2-mediated protein ubiquitination of p57. Loss of SKP2 hinders CSN6-mediated p57 destabilization, suggesting collaboration between SKP2 and CSN6 in controlling degradation of p57. Furthermore, downregulation of p57 expression by CSN6 leads to faster cell growth, deregulated cell cycle, and potentiated transformational activity [219]. However, until now, the deubiquitinase of p57 has not been reported. 

### 6.4. INK4 Proteins

The members of INK4 family, including P16^INK4a^, p15^INK4b^, p18^INK4c^, and p19^INK4d^, block the progression of the cell cycle by binding to either CDK4 or CDK6 and inhibiting the action of cyclin D [222]. Logically, the stable expression of a CKI would be incompatible with the cell cycle progression and might instead prove important for cell cycle entry or exit, and for maintaining differentiated cells in a quiescent, post-mitotic state. In general, P16^INK4a^ and p15^INK4b^ act to inhibit the cell cycle in response to various forms of oncogenic stress [223], whereas p18^INK4c^ and p19^INK4d^ are engaged in regulating organismal development [224,225,226,227]. All INK4 proteins are stably expressed except p19^INK4d^. One feature of p19^INK4d^, which appears distinct from the remaining INK4 CKIs is the pronounced periodic accumulation of its protein during the cell cycle [228,229]. Although it has been pointed out that the INK4 proteins are also degraded by proteasome, up to now, there are few reports on the post transcriptional modification of INK4 proteins [229].

p16 is encoded by the INK4a/ARF locus, which is situated on human chromosome 9p21. This locus encodes two physically linked tumor suppressor proteins, INK4a and ARF, in humans [230]. A recent study demonstrated that CSN6 associates with and downregulates p16 stability via the 20S proteasome ubiquitin-independent degradation pathway, thus promoting cell cycle progression and cancer-cell proliferation [231]. However, we do not know yet which intermediate protein mediates the degradation of p16. It is well known that the inhibitor of DNA binding protein 1 (Id1) represses p16 expression [232,233]. Previous study has shown that Smurf2 can induce p16 expression via mediating ubiquitin-degradation of Id1, providing a mechanistic link between Smurf2 and p16 during senescence [221]. At present, there are no other E3 enzymes or deubiquitinating enzymes targeting INK4 proteins, and more exploration is needed in the future.

## 7. Discussion

The UPS, through ubiquitination and deubiquitination, coordinates cell cycle progression between each phase, ensuring a unidirectional way to the cell cycle. In this review, we not only discuss the regulation of cell cycle by the two major types of ubiquitin ligases but also summarize the effects of other E3 ligases and DUBs on the cell cycle. Due to the complexity of the UPS mechanism and the diversity of cell cycle-related proteins, we only discuss the UPS regulation involved in cyclins, CDKs and CKIs here. Actually, many other kinases also play important roles in cell cycle regulation. For instance, Aurora A and PLK1 are centrosomal kinases involved in centrosome maturation and spindle assembly [234]. Wee1 is a tyrosine kinase that plays a crucial role in the G2/M cell cycle checkpoint arrest for DNA repair before mitotic entry [235]. Many E3 ligases and DUBs targeting cyclins or CKIs have been identified, but there are too few reports about E3 ligases or DUBs targeting CDKs. On the one hand, a CDK is activated by a corresponding cyclin, and the abnormal accumulation or degradation of cyclins will directly affect the activity of CDK. On the other hand, the regulation of CKIs by UPS also influences the roles of CDKs in the cell cycle. Moreover, CKIs have received increased attention, but there are few studies on INK4 protein at present, and the research on UPS regulation of INK4 protein needs to be further explored. The cell cycle is a very complex process influenced by many aspects, and there are many kinds of proteins involved in cell cycle regulation. For example, SCF^SKP2^ not only targets the degradation of cyclin B but also mediates the proteasomal degradation of CKIs including p21, p27, and p57. Both cyclin A and cyclin D are the substrates of USP2. In the regulation of cell cycle, researchers pay more attention to the tumor suppressor p53, which is the substrate protein of many E3 ligases and DUBs (Table 4). It is noteworthy that ubiquitin-mediated proteolysis plays a significant role in many peripheral cell cycle-linked processes beyond regulating major cell cycle phase transitions, such as maintenance and assembly of the mitotic spindle, chromosome condensation, DNA damage response, and centrosome duplication.

Cancer is regarded as a disease of deregulated cell proliferation. Each of the pathways that constrains the proliferative response in normal cells is perturbed in most cancers [29]. As a negative regulator of the cell cycle, targeting a CKI has always been considered as a promising strategy of cancer treatment. For CKI, the DUB that makes it stable is generally a tumor suppressor, while the E3 ligase that promotes its degradation is generally an oncoprotein, and vice versa for CDKs and cyclins. For a long time, countless studies have been devoted to finding new targets for cancer treatment. Attempts to develop proteasome inhibitors for cancer therapy have been questioned because suppression of the proteasome is likely to be highly toxic to normal cells. In addition, proteasome inhibitors have dramatically improved outcomes, but relapses are frequent and acquired resistance to treatment eventually emerges [237]. Although ubiquitination events are considered to be promising targets for cancer therapy, the identification of efficient and specific ubiquitin ligase inhibitors has not been successful due to the complexity of the enzymatic cascade involved in ubiquitin binding. However, since deubiquitination is a simpler process similar to proteolysis, DUBs may prove to be better targets to develop enzyme inhibitors for cancer therapy. 

## Figures and Tables

**Table 1:** Regulation of UPS machinery on cyclins and its functional roles in the cell cycle.

E3 Ligase/DUB	Target	Function	Reference
**E3 ligase**			
APC/C^Cdc20^ APC/C^Cdh1^	cyclin A	Promotes the onset of anaphase and exit from Mitosis	[78]
APC/C^Cdc20^	cyclin B	Inhibits B-type cyclin accumulation and to prevent uncontrolled entry into S phase	[91]
HEI10	cyclin B1	Functions in progression through G2/M	[92]
SCF^SKP2^	cyclin D	Regulates the mammalian G1/S transition	[71]
FBXW8	cyclin D1	Promotes cancer cell proliferation	[68]
FBXO25	Oct-1	Promotes tumor growth and metastasis	[69,93]
SCF^FBX4-αB crystallin^	cyclin D1	Knockdown of the SCF^FBX4-αB crystallin^ ligase accelerates G1 phase progression	[94]
Cullin-3	cyclin E1	Controls S phase in mammalian cells	[95]
SCF^Fbw7^	cyclin E	Inhibits cancer cell proliferation	[70]
SCF^SKP2^	cyclin E	Plays a role in the S phase progression	[96]
**DUB**			
USP37	cyclin A	Accelerates S phase entry	[83]
USP2a	cyclin A1	Increases cell proliferation	[84]
DUB3 (USP17)	cyclin A	Be critical for G1/S transition	[85]
Bam/Otu complex	cyclin A	Contributes to germ-line stem cell fate determination	[90]
USP14	cyclin B1	Facilitates G2/M phase transition	[97]
USP22	cyclin B1	USP22 knockdown leads to slower cell growth	[98]
OTUD7B	cyclin B	Contributes to mitotic exit	[99]
USP2	cyclin D1	Accelerates the G1 to S phase progression	[100]
USP9x	FoxO3a	Suppresses cell proliferation by downregulating expression of cyclin D	[101,102]
OTUD6B	?	Blocks cell proliferation by arresting cells in G1 phase	[103]
USP22	cyclin D1	Promotes G1/S cell cycle transition	[104]
USP27	cyclin E	Increases the percentage of cells in G2/M phase and decreases the percentage of cells in G0/G1 phase	[105]

**Table 2:** CDK and its related UPS regulation.

E3 Ligase/DUB	Target	Function	Reference
**E3 ligase**			
KLHL6	CDK2	Drives granulocytic differentiation in human AML cells	[130]
**DUB**			
Ubp-M	uH2A	Facilitates chromosome condensation and cell cycle progression	[124]
UCH-L1	CDK1/4/5	Enhances CDKs activity and promotes cell proliferation	[126]
CSN5	CDK2	Promotes cell cycle progression	[127]
DUB3	SNAIL1	Regulates cell migration and cancer metastasis	[131]

**Table 3:** Regulation of ubiquitination and deubiquitination involved in CKIs.

Substrate	E3 Ligase/DUB		Function	Reference
**E3 ligase**				
p21^CIP1/WAF1^	SCF^SKP2^	Regulates the mammalian G1/S transition		[71,72,73]
	APC/C^CDC20^	Contributes to the full activation of Cdk1 necessary for mitotic events and prevents mitotic slippage during spindle checkpoint activation		[186]
	CRL4^CDT2^	Promotes DNA repair by promoting the degradation of p21		[187,188,189]
	MKRN1	Depletion of MKRN1 induces cell cycle arrest by activating p53 and p21		[164]
	CRL2^LRR1^	Nematode CRL2^LRR1^ ensures G1-phase cell cycle progression in germ cells, whereas human CRL2^LRR−1^ has no significant effect on cell cycle		[190]
	ZNF313	Activates cell cycle progression and inhibits cellular senescence		[191]
	RNF126	Facilitates cell cycle G1/S progression and cell proliferation		[192]
	CHIP	Knockdown of CHIP results in enhanced cellular senescence and increased sensitivity of lung cancer cells to ionizing radiation		[193]
	CRL4B^DCAF11^	Promotes S phase entry and osteosarcoma cell proliferation		[194]
	UHRF2	Promotes DNA damage response		[195]
	SPSB1	SPSB1 knockdown induced cell cycle arrest and apoptosis		[196]
	NEDD4	Promotes cell growth		[197]
	FBXO22	Promotes cell growth, and affects cell cycle and apoptosis		[198]
p27^KIP1^	SCF^SKP2^	Induces S phase in quiescent cells		[74,206,207,208]
	KPC	Controls cell cycle progression from G0 to S phase		[209]
	Pirh2	Depletion of Pirh2 induces an inhibition of cell cycle progression at G1/S transition		[210]
	COP1	Cell cycle progression is delayed with COP1 deficiency		[211]
p57^KIP2^	SCF^SKP2^	Contributes to cell cycle progression		[217,220]
	FBL12	Regulates osteoblast cell differentiation		[218]
Id1	Smurf2	Provides a mechanistic link between Smurf2 and p16 during senescence		[221]
**DUB**				
p21^CIP1/WAF1^	USP11	Regulates G1/S transition and the DNA damage response		[199]
p27^KIP1^	USP37	Controls cell proliferation		[215]

**Table 4:** E3 ligases and DUBs that directly regulate the p53 protein.

E3 Ligase/DUB	Function	Reference
**E3 ligase**		
MDM2	Blocks p53-dependent transcription, and promotes the rapid degradation of p53	[140,141,142]
Pirh2	Represses p53-dependent transactivation and cell cycle arrest	[156]
ICP0	Inhibits the apoptotic response to DNA damage in irradiated U2OS cells	[157]
COP1	Inhibits p53-dependent transcription, apoptosis, and cell cycle arrest	[158]
Topors	Acts as a coactivator of p53 in response to DNA damage	[159,236]
CHIP	Influences p53-mediated transcription	[160]
ARE-BP1	Inhibits p53-dependent apoptosis	[161]
TRIM24	Inhibits p53-dependent apoptosis	[162]
HRD1	Directly regulates p53-dependent apoptotic pathway in *Drosophila* fly	[163]
MKRN1	Leads cells to p53-dependent apoptosis by suppressing p21	[164]
WWP1	Increases p53 stability, and decreases p53 transcriptional activities	[165]
TRIM39	Promotes G1/S transition and cell proliferation	[166]
TRAF7	Promotes cell proliferation	[167]
Cullin4B	Promotes cell proliferation	[168]
TRIM71	Antagonizes p53-dependent pro-apoptotic and pro-differentiation responses.	[169]
RING1	Promotes cancer cell proliferation and survival	[170]
FBW7α	Responds to DNA damage	[171]
TRIM69	Decreases cell apoptosis and ROS production after UVB irradiation	[172]
**DUB**		
HAUSP (USP7)	Plays a dual role in the regulation of p53 function	[153,154,155]
USP10	Potentiates p53-dependent transcription activity and apoptosis	[173]
Otub1	Results in apoptosis and inhibition of cell proliferation in a p53-dependent manner	[174]
USP29	Stabilizes p53 in response to oxidative stress	[175]
USP42	Contributes to the repair and recovery of cells from mild or transient damage	[176]
USP11	Promotes p53 activation in response to DNA damage	[177]
USP9x	Stabilizes p53 and increases p53-dependent apoptosis	[178]
USP24	Regulates the DNA damage response	[179,180]
Ataxin-3	Regulates the functions of p53 in transactivation and apoptosis	[181]
